# Using Tenofovir Diphosphate Levels to Evaluate Factors Associated With PrEP Non‐Adherence and Performance of Indirect Adherence Measures in Latin America: A Prospective, Single‐Arm, Open‐Label, Multicentre Implementation Study (ImPrEP)

**DOI:** 10.1002/jia2.70154

**Published:** 2026-07-25

**Authors:** Thiago S. Torres, Mayara Secco Torres Silva, Brenda Hoagland, Luana Monteiro Spindola Marins, Carolina Coutinho, Emilia M. Jalil, Pedro Leite, Marcelo Cunha, Ronaldo Moreira, Iuri C. Leite, Kelika Konda, Juan Guanira, Marcos Benedetti, Cristina Pimenta, Hamid Vega‐Ramirez, Sandra W. Cardoso, Carlos F. Caceres, Peter L. Anderson, Beatriz Grinsztejn, Valdilea G. Veloso, for ImPrEP Study Group

**Affiliations:** ^1^ Instituto Nacional de Infectologia Evandro Chagas, Fundação Oswaldo Cruz (INI‐Fiocruz) Rio de Janeiro Brazil; ^2^ Escola Nacional de Saúde Pública, Fundação Oswaldo Cruz (ENSP‐Fiocruz) Rio de Janeiro Brazil; ^3^ Universidad Peruana Cayetano Heredia, Centro de Investigaciones Interdisciplinarias en Sexualidad, SIDA y Sociedad Lima Peru; ^4^ Instituto Nacional de Psiquiatria Ramón De La Fuente Muñiz Mexico City Mexico; ^5^ University of Colorado Aurora Colorado USA

**Keywords:** adherence, HIV prevention, Latin America, pre‐exposure prophylaxis (PrEP), sexual and gender minorities, young

## Abstract

**Introduction:**

Tenofovir‐based oral pre‐exposure prophylaxis (PrEP) is pivotal for HIV prevention in Latin America. Monitoring PrEP adherence is crucial for maximizing its impact and identifying individuals who may benefit from additional support. We evaluated the performance of indirect adherence measures and factors associated with PrEP non‐adherence in Brazil, Mexico and Peru.

**Methods:**

ImPrEP was a prospective, multicentre, PrEP implementation study which enrolled 9509 persons from 6 February 2018 to 30 June 2021. For this analysis, we included men who have sex with men (MSM) aged 18–24 years and transgender women aged 18+ years with at least one dried blood spot (DBS) sample collected. Adherence was assessed objectively (tenofovir diphosphate [TFV‐DP] levels in DBS) and indirectly (medication possession rate [MPR] and self‐report). We estimated area under the curve (AUC) to evaluate the performance of each indirect adherence measure, and the optimal cut‐off points for discriminating protective TFV‐DP levels based on the Youden index. We used generalized estimating equations to identify factors associated with PrEP non‐adherence (TFV‐DP<900 fmol/punch).

**Results:**

We included 2096 participants (1692 young MSM and 404 transgender women) with 4257 DBS samples. Overall, 62.3% DBS samples showed protective TFV‐DP levels, which declined over time (68.8% at week 4 to 33.8% at week 124). Both MPR and self‐report demonstrated moderate concordance (AUC: 0.74) with no difference between them (*p* = 0.79). Optimal cut‐offs were 97.6% for MPR and 93.3% for self‐report; self‐report showed higher sensitivity (84.6%), while MPR had higher specificity (62.9%). Among young adults, PrEP non‐adherence was higher among transgender women, participants with lower education and from Peru. Among transgender women, PrEP non‐adherence was higher among those with younger age, lower education, and from Peru and Mexico. Self‐reported sexual behaviours conveying elevated HIV exposure were associated with lower PrEP non‐adherence.

**Conclusions:**

MPR and self‐report are pragmatic and clinically useful adherence monitoring tools for PrEP programmes in Latin America. Social determinants of health, particularly education, emerged as major drivers of PrEP non‐adherence among transgender women and young MSM. To maximize impact, PrEP programmes must prioritize person‐centred interventions that address stigma within health services, remove barriers to sustained engagement, strengthen health literacy and include long‐acting PrEP modalities.

## 1 Introduction

Pre‐exposure prophylaxis (PrEP) is a cornerstone to end the HIV epidemic. Daily oral PrEP with tenofovir disoproxil fumarate 300 mg and emtricitabine 200 mg (TDF/FTC) remains an important PrEP option even in the emerging long‐acting landscape. Oral TDF/FTC is particularly relevant in resource‐constrained and politically unstable settings where cost and supply security are concerns, including Latin America. Nonetheless, the real‐world effectiveness of oral PrEP is dependent on prevention‐effective adherence, which might be challenging among persons experiencing social and structural vulnerabilities [1, 2]. In a pooled analysis of 6598 individuals from 72 PrEP studies, PrEP adherence was lower among participants of younger age, Hispanic/Latinx ethnicity and transgender women [3].

In Latin America, HIV incidence continues to rise. By 2024, an estimated 2.3 million people were living with HIV in the region, with the epidemic disproportionately affecting young gay, bisexual and other men who have sex with men (MSM), *travestis* and transgender women (TGW) [4]. HIV prevalence for MSM and TGW was estimated at 13.9% and 25.9%, respectively [5, 6]. Annualized HIV incidence among MSM and TGW attending HIV testing and prevention services in Brazil and Peru was estimated at 3.9%, particularly higher among those aged 18–24 years (5.3%) [7]. This underscores the urgency of scaling effective PrEP programmes and understanding adherence patterns. In the ImPrEP study, TGW and participants aged 18–24 years had higher PrEP non‐adherence according to medication possession rate (MPR) [1].

The search for reliable, cost‐effective and scalable adherence monitoring strategies is an ongoing priority for PrEP programmes worldwide. Accurately assessing and promoting oral PrEP adherence remains a complex challenge. Traditional indirect methods of adherence assessment, such as self‐report and MPR, are susceptible to biases and may not accurately reflect actual drug exposure [8]. Objective measures, including drug concentration levels in blood or hair samples, offer greater precision, but they are costly and logistically challenging to implement in resource‐limited settings [9]. There is a paucity of data on adherence measures in real‐world contexts in Latin America. A recent systematic review including 23 studies and 6649 persons (only one study conducted in Latin America; PrEP Brasil [10]) found mixed evidence of concordance between indirect and objective PrEP adherence measures [11].

To address this gap, we conducted a secondary analysis including TGW and young MSM followed in the ImPrEP study. We aimed to evaluate: (1) concordance between two indirect adherence measures (MPR and self‐report) and consistent prevention‐effective tenofovir diphosphate (TFV‐DP ≥900 fmol/punch) levels measured in dry blood spot (DBS) samples collected during the study; (2) factors associated with PrEP non‐adherence (TFV‐DP levels <900 fmol/punch).

## 2 Methods

### 2.1 Study Design

ImPrEP was a prospective, single‐arm, open‐label, multicentre implementation study that enrolled a convenience sample of participants in Brazil (14 sites in 11 cities), Mexico (four sites in three cities) and Peru (10 sites in six cities). Eligible participants were cisgender MSM and TGW aged ≥18 years, tested HIV‐negative and reporting at least one criterion of vulnerability to HIV. Details of the study design and other results have been previously described [1, 2, 12, 13]. For this analysis, we included young adults (18−24 years) and TGW (≥18 years) with at least one DBS sample collected during the study.

Institutional review boards (IRB) at Instituto Nacional de Infectologia Evandro Chagas, Fiocruz (Brazil; #CAAE:79259517.5.1001.5262), National Institute of Public Health (Mexico; #CI‐1515) and Universidad Peruana Cayetano Heredia (Peru; #100740) approved the study. Ethical approvals were also obtained for the WHO Research Ethics Review Committee ERC and local IRB at each Brazilian site. All study participants provided written informed consent before enrolment.

### 2.2 Procedures

From 6 February 2018 to 30 June 2021, eligible participants were enrolled and received daily oral PrEP with TDF/FTC. Follow‐up visits were scheduled at week 4 and quarterly thereafter until study termination (30 June 2021). At each visit, participants received oral PrEP refills according to the next scheduled visit interval. HIV testing was conducted at every visit using rapid tests, and confirmatory testing followed each country's algorithm. We collected DBS for TFV‐DP assessments during follow‐up. TFV‐DP concentrations were measured using liquid chromatography‐mass spectrometry or mass spectrometry at the University of Colorado Antiviral Pharmacology Laboratory following standard procedures [14, 15, 16].

### 2.3 Sociobehavioural Measures

Participants completed structured questionnaires at all visits. At enrolment, we collected data on gender, age, education, race, postexposure prophylaxis use in the last 12 months, main reason to attend the service and any bacterial sexually transmitted infections (STI) diagnosis (syphilis, chlamydia or gonorrhoea). At enrolment and follow‐up visits, we collected data on the number of sex partners, condomless receptive anal sex, partner(s) living with HIV, transactional sex, stimulant drug use (e.g. ecstasy, lysergic acid diethylamide, gamma‐hydroxybutyrate), cocaine), poppers use and binge drinking (5+ drinks within 2 h). At enrolment, questions on sexual behaviour and substance use referred to the previous 6 months, except for the number of sex partners in Brazil and Mexico, which referred to the previous 3 months. At follow‐up, all questions referred to the previous 3 months.

### 2.4 Adherence Measures

We assessed PrEP adherence at follow‐up visits using indirect (MPR and self‐reported) and objective (TFV‐DP levels in DBS) adherence measures. MPR reflects the days the participant is “covered” by oral PrEP. We calculated MPR for each visit by dividing the total number of PrEP pills dispensed at the prior visit and the number of days between visits. MPR values equal or greater than 100% reflect that the participant was covered by oral PrEP during the whole period. We assessed self‐reported adherence with the question: “In the previous 30 days, how many pills did you NOT take approximately?” We provided values in percentage, with 100% reflecting no missing pills. TFV‐DP levels measured in DBS at week 4 was adjusted to account for incomplete intracellular accumulation, as the final pharmacokinetic plateau is only reached after approximately 90–100 days due to the long intracellular half‐life of TFV‐DP (∼17 days). After standardization, the same prevention‐effective TFV‐DP threshold in DBS could be applied independently of study visit (≥900 fmol/punch), equivalent to 4+ doses of PrEP per week [17].

### 2.5 Statistical Analysis

We first described the baseline characteristics of TGW and young MSM included in this analysis compared to those not included. Comparisons were performed using chi‐square or Fisher's exact test, as appropriate. We then described the number of DBS samples available per participant, number of DBS samples per week visit, and the proportion of participants with prevention‐effective TFV‐DP levels. Such descriptions were performed overall and per country for TGW and young adults (MSM and TGW).

We used generalized estimating equations (GEE) and area under the curve (AUC) using a receiver operating characteristic (ROC) to assess the accuracy of each indirect measure with protective prevention‐effective TFV‐DP levels. We performed statistical comparisons of the AUC for each adherence measure using the DeLong test, a non‐parametric method commonly used to identify differences among AUC [18]. This method does not rely on the strong normality assumptions that the alternative Binormal method does. From the ROC curves, we estimated the optimal cut‐off points for discriminating between those with or without protective TFV‐DP levels based on the Youden index, a statistical measure used to identify the optimal threshold by maximizing the combined sensitivity and specificity of indirect adherence measures compared with protective TFV‐DP levels, as well as and their respective sensitivity, specificity, negative (NPV) and positive (PPV) predictive values [19, 20, 21]. Performance analyses were performed overall and separately for young adults (18−24 years) and TGW (regardless of age).

We used GEE to assess factors associated with PrEP non‐adherence among TGW and young adults. PrEP non‐adherence was defined as TFV‐DP <900 fmol/punch. Behavioural characteristics were included as time‐varying variables. In the initial models, the effect of each variable was controlled by country and study visit. All variables statistically significant at *p*‐value ≤0.1 were included in the final adjusted model. As the analyses were restricted to participants with at least one DBS measurement, we conducted a sensitivity analysis using stabilized inverse probability weights to assess the possible influence of selection bias related to inclusion in the analytic sample [22]. We performed all analyses using SAS version 9.4 (SAS Institute, North Carolina, USA).

## 3 Results

Of 9509 persons enrolled in ImPrEP, 2871 were young MSM (18–24 years) or TGW (≥18 years). For the current analysis, we included 2096 of these participants (72.9%; *N* = 2096/2874): 1802 (72.6%; *n* = 1802/2481) young adults and 404 (74.4%; *n* = 404/543) TGW. Most of the participants included were from Brazil (*n* = 1027; 49.0%), followed by Peru (*n* = 777; 37.1%) and Mexico (*n* = 292; 13.9%). Less than 1% of participants reported no sex during the study. Compared to included participants, those excluded from this analysis were more likely to be from Mexico, not identifying as White, and reporting transactional sex, stimulant use and popper use (Table 1).

**TABLE 1:** Baseline characteristics of transgender women (aged 18+ years) and young adults (aged 18–24 years) included in this analysis compared to not included.

	Transgender women (all ages) and young MSM (18–24 years)			Young adults (18–24 years)			Transgender women (all ages)		
	Included *n* (%)	Not included *n* (%)	*p*‐value	Included *n* (%)	Not included *n* (%)	*p*‐value	Included *n* (%)	Not included *n* (%)	*p*‐value
Total	2096 (72.9)	778 (27.1)		1802 (72.6)	679 (27.4)		404 (74.4)	139 (25.6)	
**Country**			<0.0001			<0.0001			<0.0001
Brazil	1027 (49.0)	138 (17.7)		921 (51.1)	112 (16.5)		154 (38.1)	41 (29.5)	
Mexico	292 (13.9)	419 (53.9)		258 (14.3)	385 (56.7)		50 (12.4)	45 (32.4)	
Peru	777 (37.1)	221 (28.4)		623 (34.6)	182 (26.8)		200 (49.5)	53 (38.1)	
**Gender**			0.39			0.84			NA
Transgender women	404 (19.3)	139 (17.9)		110 (6.1)	40 (5.9)		404 (100.0)	139 (100.0)	
Cisgender men	1692 (80.7)	639 (82.1)		1692 (93.9)	639 (94.1)		0 (0.0)	0 (0.0)	
**Age (years)**			<0.0001			<0.0001			0.26
18−19	252 (12.0)	97 (12.5)		252 (14.0)	97 (14.3)		12 (3.0)	4 (2.9)	
20−21	540 (25.8)	130 (16.7)		540 (30.0)	130 (19.1)		37 (9.2)	10 (7.2)	
22−24	1010 (48.2)	452 (58.1)		1010 (56.0)	452 (66.6)		61 (15.1)	26 (18.7)	
25−30	109 (5.2)	48 (6.2)		0 (0.0)	0 (0.0)		109 (27.0)	48 (34.5)	
>30	185 (8.8)	51 (6.6)		0 (0.0)	0 (0.0)		185 (45.8)	51 (36.7)	
**Education**			0.13			0.12			0.16
Primary	41 (2.0)	22 (2.8)		22 (1.2)	13 (1.9)		31 (7.7)	17 (12.2)	
Secondary	681 (32.5)	229 (29.4)		518 (28.7)	172 (25.3)		229 (56.7)	81 (58.3)	
Post‐secondary	1374 (65.6)	527 (67.7)		1262 (70.0)	494 (72.8)		144 (35.6)	41 (29.5)	
**Race**			<0.0001			<0.0001			0.04
Asian, Black, Indigenous, *Pardo* or *Mestizo*	1582 (75.5)	679 (87.3)		1351 (75.0)	592 (87.2)		312 (77.2)	119 (85.6)	
White	514 (24.5)	99 (12.7)		451 (25.0)	87 (12.8)		92 (22.8)	20 (14.4)	
**Previous PEP use**			0.0032			0.0078			0.12
Yes	359 (17.1)	98 (12.6)		319 (17.7)	90 (13.3)		59 (14.6)	13 (9.4)	
No	1737 (82.9)	680 (87.4)		1483 (82.3)	589 (86.7)		345 (85.4)	126 (90.6)	
**Main reason to attend the service**			0.32			0.14			0.89
Seeking PrEP	1720 (82.1)	626 (80.5)		1530 (84.9)	560 (82.5)		270 (66.8)	92 (66.2)	
Other	376 (17.9)	152 (19.5)		272 (15.1)	119 (17.5)		134 (33.2)	47 (33.8)	
**Number of sex partners**			0.026			0.013			0.94
0−1	345 (16.5)	102 (13.1)		328 (18.2)	97 (14.3)		23 (5.7)	7 (5.0)	
2−3	466 (22.2)	159 (20.4)		429 (23.8)	146 (21.5)		46 (11.4)	15 (10.8)	
≥4	1285 (61.3)	517 (66.5)		1045 (58.0)	436 (64.2)		335 (82.9)	117 (84.2)	
**Condomless receptive anal sex**			0.026			0.022			0.66
Yes	1493 (71.2)	521 (67.0)		1244 (69.0)	436 (64.2)		349 (86.4)	118 (84.9)	
No	603 (28.8)	257 (33.0)		558 (31.0)	243 (35.8)		55 (13.6)	21 (15.1)	
**Partners living with HIV**			0.057			0.034			0.32
Yes	313 (14.9)	106 (13.6)		301 (16.7)	103 (15.2)		19 (4.7)	4 (2.9)	
No	678 (32.3)	223 (28.7)		584 (32.4)	191 (28.1)		127 (31.4)	37 (26.6)	
Don't know	1105 (52.7)	449 (57.7)		917 (50.9)	385 (56.7)		258 (63.9)	98 (70.5)	
**Transactional sex**			<0.0001			<0.0001			0.22
Yes	533 (25.4)	258 (33.2)		340 (18.9)	187 (27.5)		283 (70.0)	105 (75.5)	
No	1563 (74.6)	520 (66.8)		1462 (81.1)	492 (72.5)		121 (30.0)	34 (24.5)	
**Stimulant drug use** ^a^			<0.0001			<0.0001			0.38
Yes	254 (12.1)	152 (19.5)		204 (11.3)	131 (19.3)		79 (19.6)	32 (23.0)	
No	1842 (87.9)	626 (80.5)		1598 (88.7)	548 (80.7)		325 (80.4)	107 (77.0)	
**Poppers use**			<0.0001			<0.0001			0.09
Yes	164 (7.8)	174 (22.4)		156 (8.7)	165 (24.3)		17 (4.2)	11 (7.9)	
No	1932 (92.2)	604 (77.6)		1646 (91.3)	514 (75.7)		387 (95.8)	128 (92.1)	
**Binge drinking**			0.0027			0.0009			0.92
Yes	1513 (72.2)	517 (66.5)		1316 (73.0)	450 (66.3)		278 (68.8)	95 (68.3)	
No	583 (27.8)	261 (33.5)		486 (27.0)	229 (33.7)		126 (31.2)	44 (31.7)	
**Any bacterial STI**			0.12			0.20			0.38
Yes	615 (31.4)	187 (28.1)		514 (30.7)	160 (27.9)		146 (37.6)	42 (33.3)	
No	1345 (68.6)	478 (71.9)		1161 (69.3)	414 (72.1)		242 (62.4)	84 (66.7)	

Abbreviations: MSM, gay, bisexual and other men who have sex with men; NA, not applicable; PEP, HIV post‐exposure prophylaxis; PrEP, HIV pre‐exposure prophylaxis; STI, sexually transmitted infection.

^a^ Including ecstasy, lysergic acid diethylamide, gamma‐hydroxybutyrate and cocaine.

Over a third of participants had only one DBS sample available (757, 36.1%) (Table 2). Of all 4274 DBS samples, 2662 (62.3%) had prevention‐effective TFV‐DP levels, higher at week 4 (*n* = 1311; 68.8%) and lower at week 124 (*n* = 22; 33.8%). Among young adults, 2274 (63.6%) DBS samples had prevention‐effective TFV‐DP levels, higher at week 4 (*n* = 1160; 70.5%) and lower at week 124 (*n* = 5; 15.6%). Among TGW, 495 (53.3%) DBS samples had prevention‐effective TFV‐DP levels, 55.2% (*n* = 198) at week 4 and 47.6% (*n* = 20) at week 124.

**TABLE 2:** Number of dried blood spots (DBS) samples per participant, number of DBS samples per week visit and proportion of participants with protective tenofovir‐diphosphate (TFV‐DP) levels.^a^

Week visit	Overall	Young adults (18−24 years)				Transgender women (all ages)			
		Total	Brazil	Mexico	Peru	Total	Brazil	Mexico	Peru
**Number of DBS samples per participant (*n*; %)**									
1	757 (36.1)	661(36.7)	259 (28.1)	137 (53.1)	265 (42.5)	145 (35.9)	40 (26.0)	34 (68.0)	71 (35.5)
2	647 (30.9)	582 (32.3)	321 (34.8)	108 (41.9)	153 (24.6)	92 (22.8)	32 (20.8)	13 (26.0)	47 (23.5)
3	571 (27.2)	500 (27.8)	318 (34.5)	13 (5.0)	169 (27.1)	87 (21.5)	29 (18.8)	3 (6.0)	55 (27.5)
4	96 (4.6)	47 (2.6)	19 (2.1)	0 (0.0)	28 (4.5)	63 (15.6)	41(26.6)	0 (0.0)	22 (11.0)
5	24 (1.1)	12 (0.7)	4 (0.4)	0 (0.0)	8 (1.3)	16 (4.0)	11 (7.1)	0 (0.0)	5 (2.5)
6	1 (0.1)	0 (0.0)	0 (0.0)	0 (0.0)	0 (0.0)	1 (0.2)	1 (0.7)	0 (0.0)	0 (0.0)
**Number of DBS samples per week visit (*n*; %)**									
4	1906 (44.6)	1645 (46.0)	875 (44.8)	211 (53.8)	559 (45.4)	359 (39.5)	142 (34.1)	42 (60.9)	175 (39.5)
28	1169 (27.4)	1010 (28.3)	643 (32.9)	71 (18.1)	296 (24.1)	215 (23.2)	102 (24.5)	12 (17.4)	101 (22.8)
52	745 (17.4)	631 (17.7)	383 (19.6)	66 (16.8)	182 (14.8)	139 (15.0)	66 (15.9)	5 (7.3)	68 (15.3)
76	254 (5.9)	189 (5.3)	30 (1.5)	42 (10.7)	117 (9.5)	84 (9.0)	37 (8.9)	9 (13.0)	38 (8.6)
100	135 (3.2)	66 (1.8)	15 (0.8)	1 (0.3)	50 (4.1)	89 (9.6)	52 (12.5)	0 (0.0)	37 98.4)
124	65 (1.5)	32 (0.9)	5 (0.3)	1 (0.3)	26 (2.1)	42 (4.5)	17 (4.1)	1 (1.4)	24 (5.4)
**Total**	**4274** ^b^	**3573**	**1951**	**392**	**1230**	**928**	**416**	**69**	**443**
**Proportion of participants with protective TFV‐DP levels per week visit (*n*; %)** ^b^									
4	1311 (68.8)	1160 (70.5)	731 (83.5)	179 (84.8)	250 (44.7)	198 (55.2)	110 (77.5)	27 (64.3)	61 (34.9)
28	718 (61.4)	635 (62.9)	489 (76.0)	58 (81.7)	88 (29.7)	109 (50.7)	81 (79.4)	7 (58.3)	21 (20.8)
52	442 (59.3)	382 (60.5)	273 (71.3)	53 (80.3)	56 (30.8)	74 (53.2)	55 (83.3)	2 (40.0)	17 (25.0)
76	105 (41.3)	70 (37.0)	13 (43.3)	31 (73.8)	26 (22.2)	43 (51.2)	29 (78.4)	6 (66.7)	8 (21.0)
100	64 (47.4)	22 (33.3)	10 (66.7)	0 (0.0)	12 (24.0)	51 (57.3)	39 (75.0)	0 (0.0)	12 (32.4)
124	22 (33.8)	5 (15.6)	3 (60.0)	1 (100.0)	1 (3.8)	20 (47.6)	13 (76.5)	1 (100.0)	6 (25.0)
**Total**	**2662 (62.3)**	**2274 (63.6)**	**1519 (77.9)**	**322 (82.1)**	**433 (35.2)**	**495 (53.3)**	**327 (78.6)**	**43 (62.3)**	**125 (28.2)**

*Note*: Bold refers to total values.

^a^ TFV‐DP concentration ≥ 900 fmol/punch.

^b^ Two hundred and twenty‐seven samples refer to young transgender women.

**FIGURE 1:**
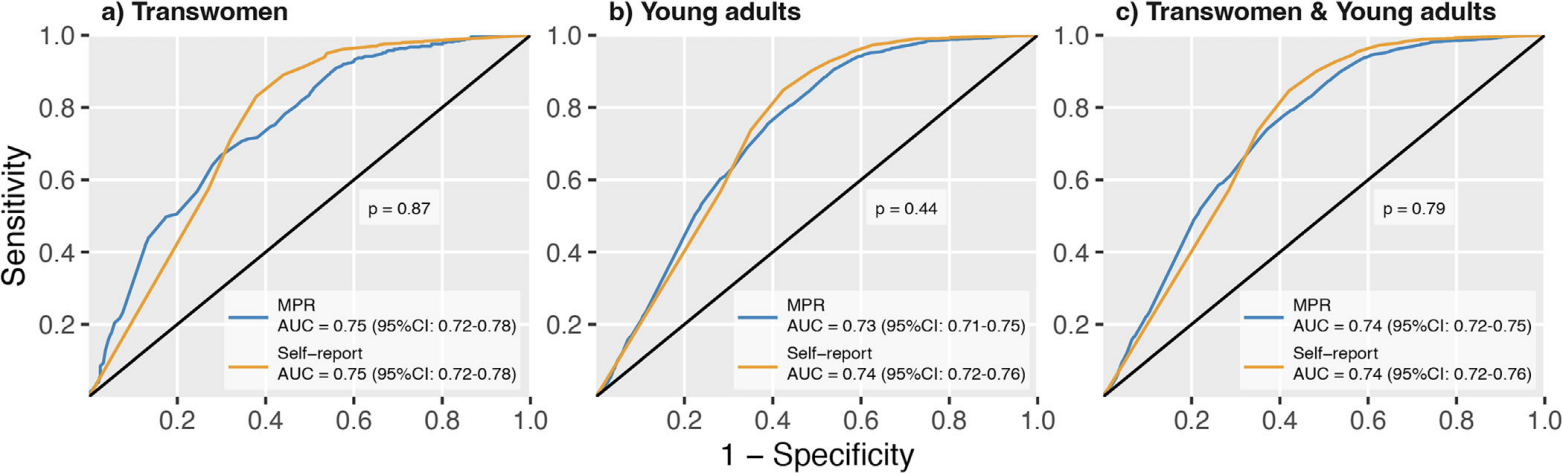
Receiver operating characteristic (ROC) curves of indirect adherence measures to predict protective tenofovir‐diphosphate (TFV‐DP) levels. *p*‐value refers to the difference between the area under the curve (AUC) of the two indirect adherence measures (De Long's test).

Overall, the area under the ROC curve was 0.74 (95% CI: 0.72−0.75) for MPR and 0.74 (95% CI: 0.72−0.76) for self‐report (Figure 1), with similar performance between young adults and TGW. The discriminatory ability of MPR and self‐report did not differ significantly (*p*>0.05). Cut‐off values associated with prevention‐effective TFV‐DP levels were 97.6% for MPR and 93.3% for self‐report (Table 3). We found the same cut‐off values for self‐report among young adults and TGW, while cut‐off values for MPR were lower for young adults (87.0%) than TGW (97.8%). Overall, self‐report demonstrated higher sensitivity (84.6%) compared to MPR (74.0%), but lower specificity (57.9%) compared to MPR (62.9%). Both methods showed similar PPV (∼76%), while NPV was higher for self‐report (69.5%) compared to MPR (59.4%).

**TABLE 3:** Association between indirect adherence measures and protective tenofovir‐diphosphate (TFV‐DP) levels.^a^

Population/measure	Cut‐off (%)	Sensitivity (%)	Specificity (%)	PPV (%)	NPV (%)
All participants					
MPR	97.6	74.0	62.9	76.7	59.4
Self‐report	93.3	84.6	57.9	76.8	69.5
Young adults (18−24 years)					
MPR	87.0	90.5	46.2	74.6	73.6
Self‐report	93.3	84.8	57.6	77.8	68.5
Transgender women (all ages)					
MPR	97.8	66.7	70.2	71.9	64.8
Self‐report	93.3	83.0	62.1	71.5	76.2

Abbreviations: MPR, medication possession rate; NPV, negative predictive values; PPV, positive predictive values.

^a^ TFV‐DP concentration ≥ 900 fmol/punch.

In adjusted models for young adults, the odds of PrEP non‐adherence were higher among participants from Peru (aOR: 6.31 [95% CI: 5.22−7.63]), TGW (aOR: 1.83 [95% CI: 1.17−2.86]) and those with primary education (aOR: 3.34 [95% CI: 1.22−9.15]) (Table 4). The odds of PrEP non‐adherence were lower among those reporting >3 sex partners (aOR: 0.68 [95% CI: 0.56−0.84]) and poppers use (aOR: 0.57 [95% CI: 0.38−0.86]).

**TABLE 4:** Factors associated with PrEP non‐adherence^a^ among transgender women (all ages) and young adults (aged 18–24 years).

	Young adults (aged 18−24 years)				Transgender women (aged 18+ years)			
	OR (95% CI)	*p*‐value	aOR (95% CI)	*p*‐value	OR (95% CI)	*p*‐value	aOR (95% CI)	*p*‐value
**Country** ^b^								
Brazil	1	—	1		1	—	1	—
Mexico	0.75 (0.56−1.02)	0.068	0.90 (0.64−1.25)	0.52	**1.96 (1.08**−**3.54)**	**0.026**	**2.28 (1.26**−**4.13)**	**0.0063**
Peru	**6.19 (5.14**−**7.46)**	**<0.0001**	**6.31 (5.22**−**7.63)**	**<0.0001**	**8.47 (5.62**−**12.77)**	**<0.0001**	**7.86 (4.72**−**13.07)**	**<0.0001**
**Gender** ^b^								
Cisgender men	1	—	1	—	NA	NA	NA	NA
Transgender women	**2.15 (1.44**−**3.23)**	**0.0002**	**1.83 (1.17**−**2.86)**	**0.0082**	NA	NA	NA	NA
**Age (transgender women)** ^b^								
18−24	NA	NA	NA	NA	**1.98 (1.21**−**3.25)**	**0.0068**	**2.06 (1.23**−**3.45)**	**0.0062**
25−30	NA	NA	NA	NA	0.88 (0.57−1.35)	0.55	0.90 (0.58−1.39)	0.64
>30	NA	NA	NA	NA	1	—	1	—
**Age (young adults)** ^b^								
18−19	1.11 (0.85−1.44)	0.45	NA	NA	NA	NA	NA	NA
20−21	0.97 (0.79−1.19)	0.76	NA	NA	NA	NA	NA	NA
22−24	1		NA	NA	NA	NA	NA	NA
**Education** ^b^								
Primary	**4.72 (1.81**−**12.31)**	**0.0015**	**3.34 (1.22**−**9.15)**	**0.019**	**4.45 (1.99**−**9.93)**	**0.0003**	**3.52 (1.63**−**7.61)**	**0.0013**
Secondary	**1.24 (1.02**−**1.51)**	**0.02**	1.12 (0.92−1.36)	0.27	**1.80 (1.19**−**2.70)**	**0.0050**	**1.63 (1.08**−**2.46)**	**0.021**
Post‐secondary	1	—	1	—	1	—	1	—
**Race** ^b^								
Asian, Black, Indigenous, *Pardo* or *Mestizo*	1.03 (0.83−1.28)	0.79	NA	NA	1.36 (0.85−2.19)	0.20	NA	NA
White	1	—	NA	NA	1	—	NA	NA
**Previous PEP use** ^b^								
Yes	0.99 (0.78−1.26)	0.95	NA	NA	0.86 (0.49−1.51)	0.60	NA	NA
No	1	—	NA	NA	1	—	NA	NA
**Main reason to attend the service** ^b^								
Seeking PrEP	0.82 (0.64−1.03)	0.11	NA	NA	**0.50 (0.31**−**0.81)**	**0.0049**	**0.56 (0.34**−**0.93)**	**0.026**
Other	1	—	NA	NA	1	—	1	—
**Number of sex partners** ^c^								
0−1	1	—	1	—	1	—	NA	NA
2−3	0.86 (0.70−1.06)	0.15	0.86 (0.70−1.06)	0.16	1.01 (0.53−1.92)	0.99	NA	NA
>3	**0.71 (0.58**−**0.87)**	**0.0009**	**0.68 (0.56**−**0.84)**	**0.0002**	0.64 (0.37−1.12)	0.12	NA	NA
**Condomless receptive anal sex** ^c^								
Yes	**0.86 (0.73**−**1.02)**	**0.08**	0.86 (0.73−1.01)	0.067	**0.65 (0.47**−**0.90)**	**0.0096**	**0.65 (0.46**−**0.92)**	**0.016**
No	1	—	1	—	1	—	1	—
**Sex with partner(s) living with HIV** ^c^								
Yes	0.87 (0.66−1.15)	0.32	NA	NA	**0.33 (0.12**−**0.95)**	**0.04**	0.35 (0.11−1.10)	0.072
No	1	—	NA	NA	1	—	1	—
Don't know	0.97 (0.80−1.18)	0.75	NA	NA	0.69 (0.46−1.02)	0.066	0.74 (0.49−1.11)	0.15
**Transactional sex** ^c^								
Yes	**1.36 (1.08**−**1.72)**	**0.0080**	1.26 (0.97−1.63)	0.082	1.15 (0.76−1.73)	0.51	NA	NA
No	1	—	1	—	1	—	NA	NA
**Stimulant drug use** ^c^, ^d^								
Yes	1.03 (0.80−1.33)	0.80	NA	NA	1.30 (0.82−2.07)	0.26	NA	NA
No	1	—	NA	NA	1	—	NA	NA
**Poppers use** ^c^								
Yes	**0.53 (0.36**−**0.79)**	**0.0020**	**0.57 (0.38**−**0.86)**	**0.0069**	0.56 (0.19−1.63)	0.28	NA	NA
No	1	—	1	—	1	—	NA	NA
**Binge drinking** ^c^								
Yes	0.90 (0.77−1.06)	0.22	NA	NA	0.80 (0.58−1.08)	0.18	NA	NA
No	1	—	NA	NA	1	—	NA	NA
**Any bacterial STI** ^b^								
Yes	0.90 (0.74−1.10)	0.31	NA	NA	1.29 (0.87−1.92)	0.21	NA	NA
No	1	—	NA	NA	1	—	NA	NA

Abbreviations: CI, confidence interval; NA, not applicable; OR, odds ratio; aOR, adjusted odds ratio; PEP, HIV post‐exposure prophylaxis; PrEP, HIV pre‐exposure prophylaxis; STI, sexually transmitted infection.

Bold: *p*<0.05.

^a^ PrEP non‐adherence was defined considering TFV‐DP concentration < 900 fmol/punch.

^b^ Baseline variables.

^c^ Time‐dependent variables.

^d^ Including ecstasy, lysergic acid diethylamide, gamma‐hydroxybutyrate and cocaine.

Among TGW, the odds of PrEP non‐adherence were higher among participants from Mexico (aOR: 2.28 [95% CI: 1.26−4.13]) and Peru (aOR: 7.86 [95% CI: 4.72−13.07]), aged 18–24 years (aOR: 2.06 [95% CI: 1.23−3.45]), and reporting primary (aOR: 3.52 [95% CI: 1.63−7.61]) and secondary education (aOR: 1.63 [95% CI: 1.08−2.46]) (Table 4). The odds of PrEP non‐adherence were lower among those seeking PrEP when attending the service (aOR: 0.56 [95% CI: 0.34–0.93]) and reporting condomless receptive anal sex (aOR: 0.65 [95% CI: 0.46–0.92]).

Sensitivity analyses showed results broadly consistent with our regression models, with small differences in the magnitude of the estimated associations (Table S1).

## 4 Discussion

This PrEP implementation study (ImPrEP), which analysed 4274 DBS samples from 2096 TGW and young MSM across three Latin American countries, provides robust evidence on the practical utility of indirect adherence monitoring in real‐world settings. To our knowledge, this is the largest DBS‐based evaluation of oral PrEP adherence from Latin America published to date. Indirect adherence measures (MPR and self‐report) demonstrated similar, moderate concordance in detecting prevention‐effective TFV‐DP levels, with self‐report tending towards higher sensitivity and MPR towards higher specificity. We identified pragmatic decision thresholds (∼97% for MPR and ∼93% for self‐report) suitable for programmatic triage and intervention targeting.

Among young adults, PrEP non‐adherence was higher among those from Peru, TGW and reporting lower education. When specifically evaluating the factors associated with PrEP non‐adherence among TGW across all age groups, those from Peru, Mexico, of younger ages and with lower educational levels were more likely to experience difficulties maintaining PrEP adherence. Conversely, behaviour factors related to higher sexual exposure, such as number of partners, condomless receptive anal sex and poppers use, were protective against PrEP non‐adherence, suggesting a heightened awareness of HIV vulnerability that encourages more consistent pill use [23]. A concerning finding was the temporal decline in protective TFV‐DP levels across follow‐up, highlighting decreasing PrEP adherence over time and underscoring the critical need for sustained, proactive adherence support throughout the PrEP care continuum.

Our analysis corroborates prior studies [8, 11, 24], demonstrating that indirect PrEP adherence measures possess the ability to distinguish participants with prevention‐effective TFV‐DP concentrations throughout follow‐up. No statistically significant differences were observed between the indirect measures in their ability to distinguish between prevention‐effective and non‐effective adherence levels. This suggests that simple, scalable approaches can reliably measure adherence in real‐world settings, even when perfect agreement with objective biomarkers is not achieved. However, care should be taken with interpretation as adherence levels must be high to reliably indicate drug protection. This underscores the need for providers to actively reinforce adherence and to apply higher thresholds when assessing whether individuals are adequately protected, an approach that has direct implications for counselling around potential periods of suboptimal or unprotected exposure. The moderate AUC range reflects the inherent limitations of indirect measures, although AUC values for self‐report and MPR were higher than previously reported in studies conducted in Brazil [8, 24]. Self‐report remains prone to social desirability bias and recall error [25], while MPR captures refill behaviour rather than actual pill intake [8]. Yet, the convergence of two independent methods and their adequate discrimination in this study support their pragmatic use in stepped‐care algorithms, as routine DBS testing is resource‐prohibitive. The comparable performance validates a monitoring strategy wherein programmes can select whichever indirect measure aligns best with their infrastructure and resources (e.g. MPR for clinics with integrated pharmacy systems, self‐report for clinic‐based visits) without sacrificing discriminatory power. In Brazil, the Medicines Logistics Control System (SICLOM) contains detailed PrEP dispensation records that could be systematically leveraged as a pragmatic, low‐burden data source for population‐level adherence assessment [26]. Given MPR showed good discriminatory ability, SICLOM data could enable real‐time adherence monitoring and programmatic triage without additional clinic‐level burden, potentially serving as a replicable model for other middle‐income countries with similar pharmacy infrastructure.

In our study, PrEP non‐adherence was higher among young adults with lower education, corroborating our previous analyses using MPR‐based adherence from the entire ImPrEP cohort (*N* = 9509) [1]. A large national study of more than 20,000 sexual and gender diverse persons in Brazil showed that younger participants had lower knowledge of HIV prevention, underscoring the need for clearer, more accessible and youth‐centred messaging [27]. Overall, these results highlight the importance of PrEP strategies that actively meet young people where they are, through inclusive communication, flexible and simplified service delivery, digital tools that resonate with their daily lives and meaningful peer‐led engagement [28, 29, 30]. Ensuring that young LGBTQIAPN+ persons can fully exercise their sexuality with safety and autonomy requires services that build health literacy, reduce stigma and expand real, practical access to prevention technologies.

We found that TGW consistently showed higher rates of PrEP non‐adherence, with disparities across countries, age and education. These results align with previous ImPrEP analyses based on self‐reported adherence [2], reinforcing the persistent structural challenges faced by TGW in the region. Geographic differences may reflect unequal access to health services, varying levels of stigma and differences in health system organization, all of which can hinder sustained PrEP use. In Peru, intranational migration and forced displacement has been described as a key determinant of TGW health [31]. In Mexico, TGW have shown a big gap (>47%) between their perceived vulnerability for HIV acquisition and their actual sexual HIV exposure [32]. Brazil has a universal health system that provides care to any person in the country, which might relate to higher adherence rates [33, 34]. Unstable housing, limited employment opportunities, strained family dynamics and reduced familiarity with health systems [35, 36, 37, 38, 39], which can disrupt both medication routines and clinic attendance, are likely more prevalent among young TGW and may further heighten their vulnerability. Lower educational levels further compound these barriers by reducing health literacy and limiting the ability to navigate preventive services [40]. This finding is consistent with a previous study among TGW in Brazil, in which fewer years of schooling were associated with PrEP non‐adherence based on TFV‐DP levels [41]. These intersecting social and structural conditions reinforce the heightened vulnerability of TGW. Strengthening the role of peer educators, expanding flexible and low‐barrier delivery models and fostering supportive community networks are key components of this approach [42].

A temporal decline in protective TFV‐DP levels was observed across follow‐up, although the smaller number of samples at later visits limits the strength of conclusions regarding long‐term trends. Still, the pattern suggests a gradual reduction in adherence over time, which may reflect PrEP fatigue or persistent structural barriers to continuous engagement. Gaps between refills can disrupt medication‐taking routines, particularly when transportation or work schedules change. Furthermore, a daily oral regimen may not suit the dynamic profiles and preferences of TGW and young MSM [43]. Some individuals may moderate their use of daily oral PrEP based on changing perceptions of HIV vulnerability over time.

We found country‐level differences in PrEP non‐adherence, which may be explained by the impact of the COVID‐19 pandemic on the study. A substantial portion of the follow‐up period coincided with pandemic‐related restrictions. Although MSM and TGW were highly affected by the pandemic in Brazil, including through reduced access to HIV prevention services [44], the country was comparatively less affected due to the availability of telehealth procedures and HIV self‐testing distribution [45, 46], which helped avoid disruptions in PrEP delivery. Other factors that may explain such disparities include differences in participants’ characteristics, public health systems and awareness or availability of PrEP across countries [12, 47].

This study has limitations. ImPrEP enrolled a convenience sample across Brazil, Mexico and Peru, and for this analysis, we included only TGW (all ages) and MSM aged 18–24 years with at least one DBS sample. This may introduce selection bias and limit generalizability, as more participants from Mexico and some racial/ethnic groups were excluded from this sample. However, our results are aligned and corroborate previous analyses within the ImPrEP study using MPR and self‐report as measures of adherence [1, 2]. Furthermore, it is important to acknowledge that the analysed data reflect the period from 2018 to 2021. Despite the age of the data, our findings remain highly relevant since oral TDF/FTC is the primary PrEP modality in Latin America, while access to long‐acting PrEP is still limited. Regarding indirect adherence measures, MPR reflects refill behaviour rather than PrEP ingestion, and self‐report is susceptible to social desirability and recall biases. DBS thresholds, sampling intervals and laboratory variability may introduce measurement error, and declining participation at later visits could bias long‐term adherence estimates. While participants were prescribed and instructed to follow a daily oral PrEP regimen, we did not collect data on event‐driven dosing. Event‐driven PrEP was formally recommended by the Brazilian and Mexican Ministries of Health in 2022, and no guidelines were issued by the Peruvian Ministry of Health during the study. Its unmeasured use could potentially lead to a misclassification of adherence when compared to the daily‐dosing benchmarks used for TFV‐DP thresholds. As reports of no sex during the study were uncommon, we did not perform evaluations of adherence measures considering such periods. Lastly, our study did not measure structural factors such as stigma or discrimination. Therefore, we could not evaluate the association of these factors with PrEP non‐adherence.

Our results support an adherence monitoring algorithm wherein programmes may screen with self‐report at each visit and corroborate with MPR where feasible. Embedding these measures into electronic systems may generate real‐time alerts for clinicians, pharmacists, counsellors and other providers, enabling timely intervention. Programmes can recalibrate thresholds locally over time, emphasizing NPV during high‐capacity periods or PPV when resources are limited. These systems could be adapted for long‐acting modalities to include alerts for missing or late injection doses. For instance, during the ImPrEP CAB Brasil study, automatic alert messages were sent to pharmacists in case of late cabotegravir injection [48].

## 5 Conclusions

This large multicounty PrEP implementation study demonstrated that MPR and self‐report are pragmatic and clinically useful routine adherence monitoring tools for PrEP programmes in Latin America. The combination of MPR and self‐report assessments can help to identify individuals who may benefit from enhanced adherence support and counselling. For other PrEP formulations, such as long‐acting injectable PrEP, alternative adherence measures, including clinic visit attendance and on‐time injection receipt, should be considered.

Our findings reinforce prior evidence from ImPrEP that TGW and young MSM face greater challenges in sustaining PrEP adherence, with education playing a central role. These patterns must be viewed within the broader context of Latin America, where deep social inequities, homophobia, transphobia and the rise of anti‐gender ideologies continue to undermine access to sexual health information and services. Together, these results underscore that strengthening PrEP programmes and expanding PrEP access in the region will require more than expanding biomedical options. Effective prevention will depend on integrating person‐centred differentiated service delivery, peer and community‐based support and comprehensive sexual health education. These efforts should include enhanced counselling and education efforts to ensure individuals understand the central role of adherence. Health systems must prioritize structural adaptations to decrease barriers to access, such as the decentralization of care and the creation of stigma‐free services that integrate HIV prevention with broader social and health needs. Only by tackling these underlying determinants can PrEP, whether daily or long‐acting, reach its full potential in reducing HIV incidence.

## Author Contributions

VGV, CFC, BG, HV‐R, and BH conceived and designed the ImPrEP study. TST, MSTS, CC, BG and VGV conceived and supervised this analysis. RM, PL, MC and ICL had access to, and verified, the data. RM, PL and ICL did the statistical analyses. PLA supervised DBS analysis. TST drafted the manuscript. LMSM, EMJ, KK, JG, MB, CP and SWC helped with data acquisition and interpretation of the findings. All authors were involved in revising the manuscript for important intellectual content. All authors read and approved the final manuscript.

## ImPrEP Study Group

### Brazil

Alessandro Farias, Marcus Vinícius de Lacerda, José Valdez Madruga, Josué N. Lima, Ronaldo Zonta, Lilian Lauria, J. David Urbaez‐Brito, Polyana d'Albuquerque, Claudio Palombo, Paulo Ricardo de Alencastro, Raquel Keiko de Luca Ito, João L· de Benedetti, Fabio V· Maria, Paula M· Luz, Lucilene Freitas, Kim Geraldo, Monica Derrico, Sandro Nazer, Tania Kristic, Renato Girade (in memoriam), Renato Lima, Antônio R· de Carvalho, Carla Rocha, Pedro Leite, Marcio Lessa, Marilia Santini, Daniel R· B· Bezerra, Cleo de Oliveira Souza, Jacinto Corrêa, Marcelo Alves, Carolina Souza, Camilla Portugal, Mônica dos Santos Valões, Gabriel Lima Mota, Joyce Alves Gomes, Cynthia Ferreira Lima Falcão, Fernanda Falcão Riberson, Luciano Melo, Talita Andrade Oliva, Agnaldo Moreira de Oliveira Júnior, Bruna Fonseca, Leonor Henriette de Lannoy, Ludymilla Anderson Santiago Carlos, João Paulo da Cunha, Sonia Maria de Alencastro Coracini, Thiago Oliveira Rodrigues, Emília Regina Scharf Mettrau, Kelly Vieira Meira; Heder Tavares, Ana Paula Nunes Viveiros Valeiras, Taiane Miyake Alves de Carvalho Rocha, Alex Amorim, Patrícia Sabadini, Luiz Gustavo Córdoba; Caio Gusmão, Erika Faustino, Julia Soares da Silva Hansen, Agatha Mirian Cunha, Neuza Uchiyama Nishimura, Jaime Eduardo Flygare Razo Prereira dos Santos, Aline Barnabé Cano, Willyam Magnum Telles Dias, Magô Tonhon, Tania Regina Rezende, Alex Gomes, Eloá dos Santos Rodrigues, Maria das Dores Aires Carneiro, Alexandre Castilho, Mariana Carvalho.

### Mexico

Sergio Bautista‐Arredondo, Heleen Vermandere, Steven Díaz, Dulce Diaz‐Sosa, Centli Guillén, Lorena Hernández, Rebeca Robles, Maria Elena Medina‐Mora, Marcela González, Ivonne Huerta Icelo, Araczy Martinez Davalos, José Gomez Castro, Luis Obed Ocampo Valdez, Fernanda Ramírez Barajas, Verónica Ruiz González, Galileo Vargas Guadarrama, Israel Macías, Jehovani Tena Sánchez, Juan Pablo Osuna Noriega, H. Rodrigo Moheno, Jorge M. Bernal Ramírez, Víctor Dante Galicia Juarez, Gerardo Vizcaíno, Francisco Javier Arjona.

### Peru

Cesar Vidal Osco Tamayo, Hector Javier Salvatierra Flore, Yovanna Margot Cabrera Santa Cruz, Ricardo Martín Moreno Aguayo, Gino Calvo, Silver Vargas, Oliver Elorreaga, Ximena Gutierrez, Fernando Olivos, Damaris Caviedes, Daniella Adriazola, Eduardo Juárez, Gabriela Mariño, Jazmin Qquellon, Francesca Vasquez, Jean Pierre Jiron, Sonia Flores, Karen Campos.

## ImPrEP Study Sites

### Brazil

Fundação de Medicina Tropical (Manaus, Amazonas), Hospital Universitário Oswaldo Cruz (Recife, Pernambuco), CEDAP—Centro Estadual Especializado em Diagnóstico, Assistência e Pesquisa (Salvador, Bahia), Hospital Dia Asa Sul (Brasília, Distrito Federal), Instituto Nacional de Infectologia Evandro Chagas, Fundação Oswaldo Cruz INI‐Fiocruz (Rio de Janeiro), Hospital Municipal Rocha Maia (Rio de Janeiro), Hospital Municipal Carlos Tortelly (Niterói, Rio de Janeiro), Centro de Referência em DST/AIDS—AMDA (Campinas, São Paulo), Centro de Referência e Treinamento em DST/AIDS—CRT‐SP (São Paulo), SAE DST/AIDS—CECI (São Paulo), SAE DST/AIDS—Fidélis Ribeiro (São Paulo), SAE Adulto (Santos, São Paulo), Poli Centro (Florianópolis, Santa Catarina), SAT—Sanatório Partenon (Porto Alegre, Rio Grande do Sul).

### Mexico

Clínica Especializada Condesa (Cuauhtémoc, Mexico City), Fundación Unidos por un México Vivo A.C. (Cuauhtémoc, Mexico City), Comité Humanitario de Esfuerzo Compartido Contra El Sida A.C. (Guadalajara, Jalisco), Solidaridad Ed Thomas A·C· (Puerto Vallarta, Jalisco).

### Peru

Centro de Referencia de Infecciones de Transmisión Sexual del Centro Materno Infantil San José (Lima), Centro de Referencia de Infecciones de Transmisión Sexual del Centro Materno Infantil Tahuantinsuyo Bajo (Lima), Centro de Referencia de Infecciones de Transmisión Sexual del Centro de Salud Alberto Barton (Callao), Centro de Referencia de Infecciones de Transmisión Sexual de Caja de Agua (Lima), Centro de Referencia de Infecciones de Transmisión Sexual del Hospital Amazónico Pucallpa (Ucayali), Centro de Referencia de Infecciones de Transmisión Sexual del Hospital La Caleta Chimbote (Ancash), Centro de Referencia de Infecciones de Transmisión Sexual del Hospital Regional Ica (Ica), Centro de Referencia de Infecciones de Transmisión Sexual del Hospital Regional Trujillo (La Libertad), Investigaciones Médicas en Salud, INMENSA (Lima), Centro de Referencia de Infecciones de Transmisión Sexual del Hospital San Juan De Dios, Pisco (Ica).

## Conflicts of Interest

The authors declare no conflicts of interest.

## Supporting information




**Table S1**. Factors associated with PrEP non‐adherence among young adults aged 18–24 years and transgender women (all ages) in the adjusted and inverse probability weighted models.

## Data Availability

A complete de‐identified dataset sufficient to reproduce study findings will be made available upon request to the corresponding author, following approval of a concept sheet summarizing the analyses to be done.
