# Supplementary material for: Using Tenofovir Diphosphate Levels to Evaluate Factors Associated With PrEP Non‐Adherence and Performance of Indirect Adherence Measures in Latin America: A Prospective, Single‐Arm, Open‐Label, Multicentre Implementation Study (ImPrEP)
Source: J Int AIDS Soc. 2026 Jul 25;29(Suppl 3):e70154. doi: 10.1002/jia2.70154 (PMC13401721; doi:10.1002/jia2.70154)
Supplement: Supplementary file 1 — Table S1. Factors associated with PrEP non‐adherence among young adults aged 18–24 years and transgender women (all ages) in the adjusted and inverse probability weighted models. [file JIA2-29-e70154-s001.docx]

**Supplementary Table S1. Factors associated with PrEP nonadherence among young adults aged 18–24 years and transgender women (all ages) in the adjusted and inverse probability weighted models**

|  | **Young (18-24 years) adults** | | | | **Transgender women** | | | |  |
| --- | --- | --- | --- | --- | --- | --- | --- | --- | --- |
|  | **aOR (95% CI)** | **p-value** | **IPW aOR (95% CI)** | **p-value** | **aOR (95% CI)** | **p-value** | **IPW aOR (95% CI)** | **p-value** | |
| **Country^2^** |  |  |  |  |  |  |  |  | |
| Brazil | 1 | - | 1 | - | 1 | - | 1 | - | |
| Mexico | 0.90 (0.64-1.25) | 0.52 | 1.00 (0.71-1.39) | 0.98 | **2.28 (1.26-4.13)** | **0.0063** | **2.42 (1.34-4.34)** | **0.0032** | |
| Peru | **6.31 (5.22-7.63)** | **<0.0001** | **6.40 (5.28-7.76)** | **<0.0001** | **7.86 (4.72-13.07)** | **< 0.0001** | **8.30 (4.94-13.92)** | **<0.0001** | |
| **Gender^2^** |  |  |  |  |  |  |  |  | |
| Cisgender men | 1 | - |  |  | NA | NA | NA | NA | |
| Transgender women | **1.83 (1.17-2.86)** | **0.0082** | **2.41 (1.49-3.91)** | **0.0004** | NA | NA | NA | NA | |
| **Age (transgender women)^2^** |  |  |  |  |  |  |  |  | |
| 18-24 | NA | NA | NA | NA | **2.06 (1.23-3.45)** | **0.0062** | **2.18 (1.31-3.65)** | **0.0028** | |
| 25-30 | NA | NA | NA | NA | 0.90 (0.58-1.39) | 0.64 | 0.90 (0.58-1.39) | 0.63 | |
| >30 | NA | NA | NA | NA | 1 | - | 1 | - | |
| **Age (young adults)**^2^ |  |  |  |  |  |  |  |  | |
| 18-19 | NA | NA | NA | NA | NA | NA | NA | NA | |
| 20-21 | NA | NA | NA | NA | NA | NA | NA | NA | |
| 22-24 | NA | NA | NA | NA | NA | NA | NA | NA | |
| **Education^2^** |  |  |  |  |  |  |  |  | |
| Primary | **3.34 (1.22-9.15)** | **0.019** | **2.57 (0.98-6.74)** | **0.054** | **3.52 (1.63-7.61)** | **0.0013** | **3.60 (1.68-7.71)** | **0.0010** | |
| Secondary | 1.12 (0.92-1.36) | 0.27 | 1.16 (0.94-1.42) | 0.17 | **1.63 (1.08-2.46)** | **0.021** | **1.72 (1.14-2.60)** | **0.0097** | |
| Post-secondary | 1 | - | 1 | - | 1 | - | 1 | - | |
| **Race^2^** |  |  |  |  |  |  |  |  | |
| Asian, Black, Indigenous, *Pardo* or *Mestizo* | NA | NA | NA | NA | NA | NA | NA | NA | |
| White | NA | NA | NA | NA | NA | NA | NA | NA | |
| **Previous PEP use^2^** |  |  |  |  |  |  |  |  | |
| Yes | NA | NA | NA | NA | NA | NA | NA | NA | |
| No | NA | NA | NA | NA | NA | NA | NA | NA | |
| **Main reason to attend the service^2^** |  |  |  |  |  |  |  |  | |
| Seeking PrEP | NA | NA | NA | NA | **0.56 (0.34-0.93)** | **0.026** | **0.59 (0.35-0.98)** | **0.040** | |
| Other | NA | NA | NA | NA | 1 | - | 1 | - | |
| **Number of sex partners^3^** |  |  |  |  |  |  |  |  | |
| 0-1 | 1 | - | 1 | - | NA | NA | NA | NA | |
| 2-3 | 0.86 (0.70-1.06) | 0.16 | 0.83 (0.67-1.04) | 0.11 | NA | NA | NA | NA | |
| >3 | **0.68 (0.56-0.84)** | **0.0002** | **0.66 (0.53-0.82)** | **0.0002** | NA | NA | NA | NA | |
| **Condomless receptive anal sex^3^** |  |  |  |  |  |  |  |  | |
| Yes | 0.86 (0.73-1.01) | 0.067 | 0.85 (0.71-1.01) | 0.061 | **0.65 (0.46-0.92)** | **0.016** | **0.60 (0.41-0.87)** | **0.0076** | |
| No | 1 | - | 1 | - | 1 | - | 1 | - | |
| **Sex with partners living with HIV^3^** |  |  |  |  |  |  |  |  | |
| Yes | NA | NA | NA | NA | 0.35 (0.11-1.10) | 0.072 | 0.33 (0.11-1.00) | 0.051 | |
| No | NA | NA | NA | NA | 1 | - | 1 | - | |
| Don’t know | NA | NA | NA | NA | 0.74 (0.49-1.11) | 0.15 | 0.73 (0.48-1.10) | 0.13 | |
| **Transactional sex ^3^** |  |  |  |  |  |  |  |  | |
| Yes | 1.26 (0.97-1.63) | 0.082 | 1.24 (0.94-1.64) | 0.13 | NA | NA | NA | NA | |
| No | 1 | - |  |  | NA | NA | NA | NA | |
| **Stimulant drug use^3,4^** |  |  |  |  |  |  |  |  | |
| Yes | NA | NA | NA | NA | NA | NA | NA | NA | |
| No | NA | NA | NA | NA | NA | NA | NA | NA | |
| **Poppers use^3^** |  |  |  |  |  |  |  |  | |
| Yes | **0.57 (0.38-0.86)** | **0.0069** | **0.54 (0.34-0.83)** | **0.0055** | NA | NA | NA | NA | |
| No | 1 | - |  |  | NA | NA | NA | NA | |
| **Binge drinking^3^** |  |  |  |  |  |  |  |  | |
| Yes | NA | NA | NA | NA | NA | NA | NA | NA | |
| No | NA | NA | NA | NA | NA | NA | NA | NA | |
| **Any bacterial STI^2^** |  |  |  |  |  |  |  |  | |
| Yes | NA | NA | NA | NA | 1.29 (0.87-1.92) | 0.21 | NA | NA | |
| No | NA | NA | NA | NA | 1 | - | NA | NA | |

CI: confidence interval; NA: not applicable; aOR: adjusted odds ratio; IPW aOR: adjusted odds ratio from the inverse probability weighted model; PEP: HIV post-exposure prophylaxis; PrEP: HIV pre-exposure prophylaxis; STI: sexually transmitted infections. Bold: p<0.05; ^1^PrEP nonadherence was defined considering TFV-DP concentration < 900 fmol/punch; ^2^Baseline variables; ^3^Time-dependent variables; ^4^Including ecstasy, lysergic acid diethylamide [LSD], gamma-hydroxybutyrate [GHB]), cocaine
